# The murine Irs1 knockout model, generated by the spontaneous Irs1^S57X^ mutation in homozygosity, exhibits high embryonic lethality, limiting studies of adult hematopoiesis

**DOI:** 10.1016/j.htct.2026.106465

**Published:** 2026-04-27

**Authors:** Bruna Alves Fenerich, Jaqueline Cristina Fernandes, Cleide Lúcia Araújo Silva, João Agostinho Machado-Neto, Fabiola Traina

**Affiliations:** a Department of Medical Images, Hematology, and Oncology, University of São Paulo at Ribeirão Preto Medical School, Ribeirão Preto, São Paulo, Brazil; b Regional Blood Center of Ribeirão Preto, University of São Paulo at Ribeirão Preto Medical School, Ribeirão Preto, São Paulo, Brazil; c Department of Pharmacology, Institute of Biomedical Sciences of the University of São Paulo, São Paulo, Brazil

**Keywords:** Insulin receptor substrate 1, Knockout mice, Fetal lethality, Hematopoiesis

## Abstract

**Introduction:**

Insulin Receptor Substrate 1 (IRS1) is differentially expressed in hematological neoplasms suggesting a role in hematopoiesis and neoplastic transformation. Irs1 knockout mice represent a tool to investigate IRS1 function. This study compared hematological parameters of wild-type and heterozygous Irs1^S57X^ mice and assessed fetal lethality in homozygous mice.

**Methods:**

Hematological parameters were analyzed monthly in wild-type and heterozygous mice from 8 to 22 weeks of age. Successive intercrosses failed to yield homozygous knockouts. Fetal lethality was evaluated through timed matings of heterozygous mice, with genotyping performed at various gestational stages (E9.5, E12.5, E15.5 and E18.5).

**Results:**

Heterozygous mice showed no significant differences in body weight or hematological parameters compared with wild-type mice (all p-value >0.05). Homozygous Irs1^S57X^ mice exhibited fetal or postnatal lethality as fetuses developed until gestational stage E18.5 but were either aborted or died shortly after birth.

**Conclusion:**

The Irs1^S57X^ mutation in heterozygosis does not alter phenotype, whereas homozygosity for Irs1^S57X^ is associated with markedly reduced perinatal survival, precluding adult hematopoiesis studies. Future research should focus on fetal hematopoiesis.

## Introduction

The insulin receptor substrate (IRS) family encompasses six cytoplasmic adaptors that regulate numerous cellular processes such as growth, metabolism, survival, and proliferation [1]. Insulin Receptor Substrate 1 (IRS1) is differentially expressed in hematological neoplasms, suggesting its participation in hematopoiesis and neoplastic transformation [2, 3, 4, 5]. IRS1 acts as a central adaptor in signal transduction downstream of the insulin and Insulin-like growth factor 1 (IGF1R/IR) receptors, promoting activation of the phosphoinositide 3-kinase (PI3K)-Protein kinase B (AKT)-Mechanistic target of rapamycin (mTOR) signaling cascade [1,6]. Upon ligand binding, IRS1 is phosphorylated on tyrosine residues, creating docking sites for the p85 regulatory subunit of PI3K, leading to PIP3 production and the ensuing recruitment and activation of AKT. Subsequent activation of mTOR, particularly through the mTORC1 complex, stimulates protein synthesis, cell growth, metabolic reprogramming, and inhibition of apoptosis [1,6]. In hematologic malignancies, hyperactivation of this pathway, driven by IRS1 overexpression, autocrine/paracrine IGF1 stimulation, or upstream genetic alterations, contributes to enhanced proliferation, survival, and therapeutic resistance, as well as to metabolic adaptation and interactions with the bone marrow microenvironment [5,7, 8, 9]. Thus, IRS1 not only amplifies mitogenic and anti-apoptotic signals but also integrates metabolic and oncogenic cues, positioning it as a potential therapeutic target and biomarker in leukemias and other hematologic cancers. The role of IRS1 in the BCR::ABL1 signaling pathway was first demonstrated by our research group [1,3,10], however the hypothesis of the participation of IRS1 in normal hematopoiesis has not yet been elucidated.

In this scenario, using a murine Irs1 knockout model represents an interesting tool to evaluate the function of Irs1 in normal hematopoiesis. IRS knockout mice have been extensively used for insulin signaling pathway research, particularly in studies related to diabetes, obesity, and development. Irs1 knockout models were generated and first described by two independent research groups in 1994 [11,12].

B6.129S2-Irs1^smla^ mice carry a spontaneous nonsense mutation in serine 57 (Irs1^S57X^), which in homozygosis produces a knockout animal for Irs1, characterized by low weight and body size. This mutation arose spontaneously in an embryonic stem (ES) cell subclone during the generation of a *SerpinB2*-targeted line. Following the identification of a recombination event, the mutation was segregated from the *SerpinB2* locus, mapped to a 2.78-Mb proximal interval, and backcrossed onto a C57BL/6 background. The original phenotype included reduced growth, decreased survival, and smaller body size at birth, and the colony has since been maintained under controlled breeding conditions to ensure stable transmission of the Irs1^S57X^ allele [13]. The present study aimed to compare the hematological parameters of wild-type and heterozygous mice for the Irs1^S57X^ mutation and to describe the fetal lethality of homozygous mice.

## Material and methods

### Animals

The protocols for mouse experiments were approved by the Animal Ethics Committee of the Institution. Mice were maintained on a 12:12-h light-dark cycle and housed at the Laboratory for Experimental Animal Studies located at the Regional Blood Center Medical School, Hemotherapy Center of Ribeirão Preto. B6.129S2-Irs1^smla^/J mice (Jackson Laboratory, stock number: 007240) in a pure C57BL/6 background (≥10 generations) were mated to produce homozygous animals. Genotyping was performed by Sanger sequencing in an ABI 3500xL Genetic Analyzer (Applied Biosystems, Foster City, CA, USA).

### Hematological parameters evaluation

For peripheral blood collection, mice were anesthetized with 2 % isoflurane via inhalation. Blood samples were collected into heparinized tubes and analyzed using an automated hematology analyzer. Evaluated parameters included the white blood cell count (WBC), red blood cell count (RBC), hemoglobin level (Hb), hematocrit (Hct), and platelets. Animals were assessed monthly across four age ranges (8–10, 12–14, 16–18, and 20–22 weeks), with body weight recorded immediately prior to blood collection.

### Fetal lethality experiments

Mating was performed between heterozygous mice. Females were followed up daily, in the morning, to check for the presence of a vaginal plug. The time that plug is identified is considered as 0.5 days post coitus (dpc) or E0.5, since coitus occurs at night [14]. Once the plug was found, the females were accommodated separately until the date of euthanasia. Euthanasia of the females was performed on gestational days E9.5 (*n* = 1), E12.5 (*n* = 1), E15.5 (*n* = 2), and E18.5 (*n* = 2). The fetuses were carefully separated from the maternal material to avoid contamination in the genotyping. Throughout the development of this research, we noticed that the successive mating between Irs1^smla^ heterozygous animals did not result in homozygous mice among the offspring.

### Offspring genotyping

For early developmental stages (E9.5 and E12.5), DNA was extracted from the entire fetus, whereas for E15.5 and newborns (E18.5), DNA was obtained from the caudal portion. For extraction, 200–400 µL of NaOH (50 µM) was added to each sample, which was then incubated in a dry bath at 98 °C for 60 min (E9.5 and E12.5) for 80 min (E15.5 and E18.5). Subsequently, 50–100 µL of Tris–HCl (1 M, pH 6.8) was added to neutralize the samples. Genotyping was performed via Sanger sequencing.

## Statistical analysis

Statistical analyses were performed using GraphPad Prism 8 (GraphPad Software Inc.). Differences between groups were analyzed via two-way ANOVA with Tukey’s post-test, while Chi-square analysis was used to determine if offspring ratios deviated from Mendelian expectations. All p-values were two-sided, and the significance level was set at 5 %.

## Results

### The irs1^s57x^ spontaneous mutation, in heterozygous status, did not modulate hematological parameters and body weight

**Figure 1:**
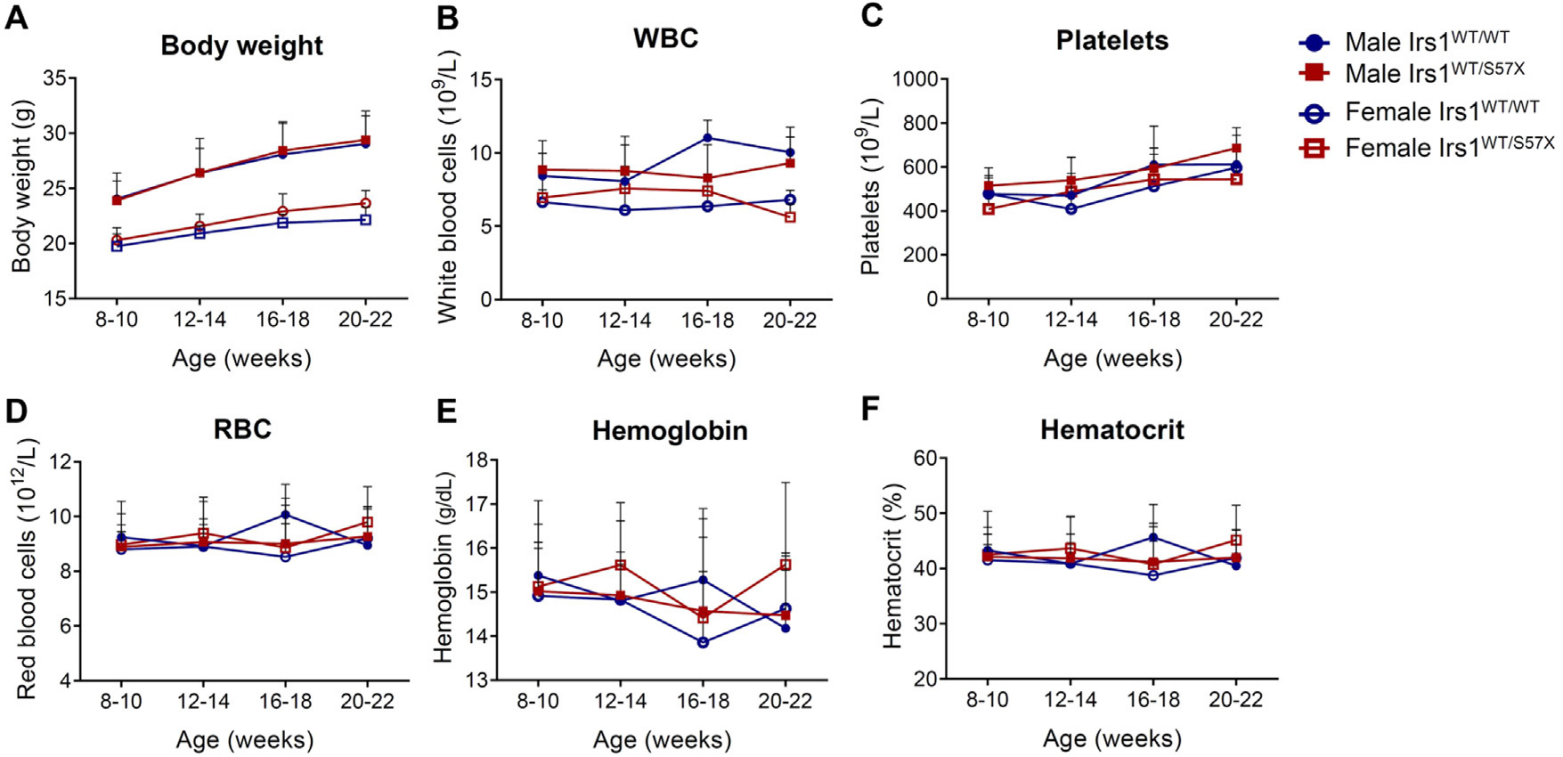
Body weight and peripheral blood hematological profile in Irs1 mice: serial evaluation. (A) Body weight. (B) White blood cell count (WBC - 10^9^/L). (C) Platelets (10^9^/L). (D) Red blood cell count (RBC - 10^12^/L). (E) Hemoglobin level (Hb - g/dL). (F) Hematocrit (Hct - %). Two-way ANOVA test and Tukey post-test; p-values are indicated in the graphs. Irs1^WT/WT^ (wild-type), Irs1^WT/S57X^ (heterozygous).

After 28 intercross events, a total of 101 heterozygotes, 46 wild-type mice and 1 homozygous mouse were obtained, with an average of 4.9 (± 1.6) born alive per female. The absence of homozygous knockout mice in the offspring precluded the inclusion of this genotype in the statistical analysis. The single Irs1 knockout mouse that survived the weaning was euthanized at nine weeks of age for analysis. The Irs1-deficient mouse was male and characterized by low body weight (15.64 *g*; WBC: 6.8 × 10^9^/L; RBC: 9.79 × 10^12^/L; Hb: 16.4 g/dL; Hct: 48.5 % and platelets: 311 × 10^9^/L). Wild-type (Irs1^WT^^/^^WT^) and heterozygous (Irs1^WT^^/S57X^) mice were monitored for four months starting at eight weeks of age with body weight and peripheral blood parameters being recorded monthly. On comparing the wild type animals (*n* = 7 females and 4 males) and heterozygotes (*n* = 7 females and 12 males), the body weight and peripheral blood hematological parameters did not differ significantly at any of the ages evaluated (all p-value *>*0.05) (Figure 1).

Since all matings were performed between heterozygous animals, a Mendelian segregation ratio of 1:2:1 was expected. Considering the total number of offspring obtained from intercrosses (*n* = 148), the expected frequencies would be 37 wild-type, 74 heterozygous, and 37 homozygous mice. However, the observed distribution (46 wild-type, 101 heterozygous, and 1 homozygous) markedly deviated from the expected ratio. Chi-square analysis demonstrated a highly significant deviation from Mendelian proportions (χ² = 39.25; df = 2; p-value *<*0.0001), indicating a marked underrepresentation of homozygous animals at birth.

### Homozygous fetuses developed until day E18.5; however, with the exception of a single pup, all were identified as aborted or died immediately after birth

**Figure 2:**
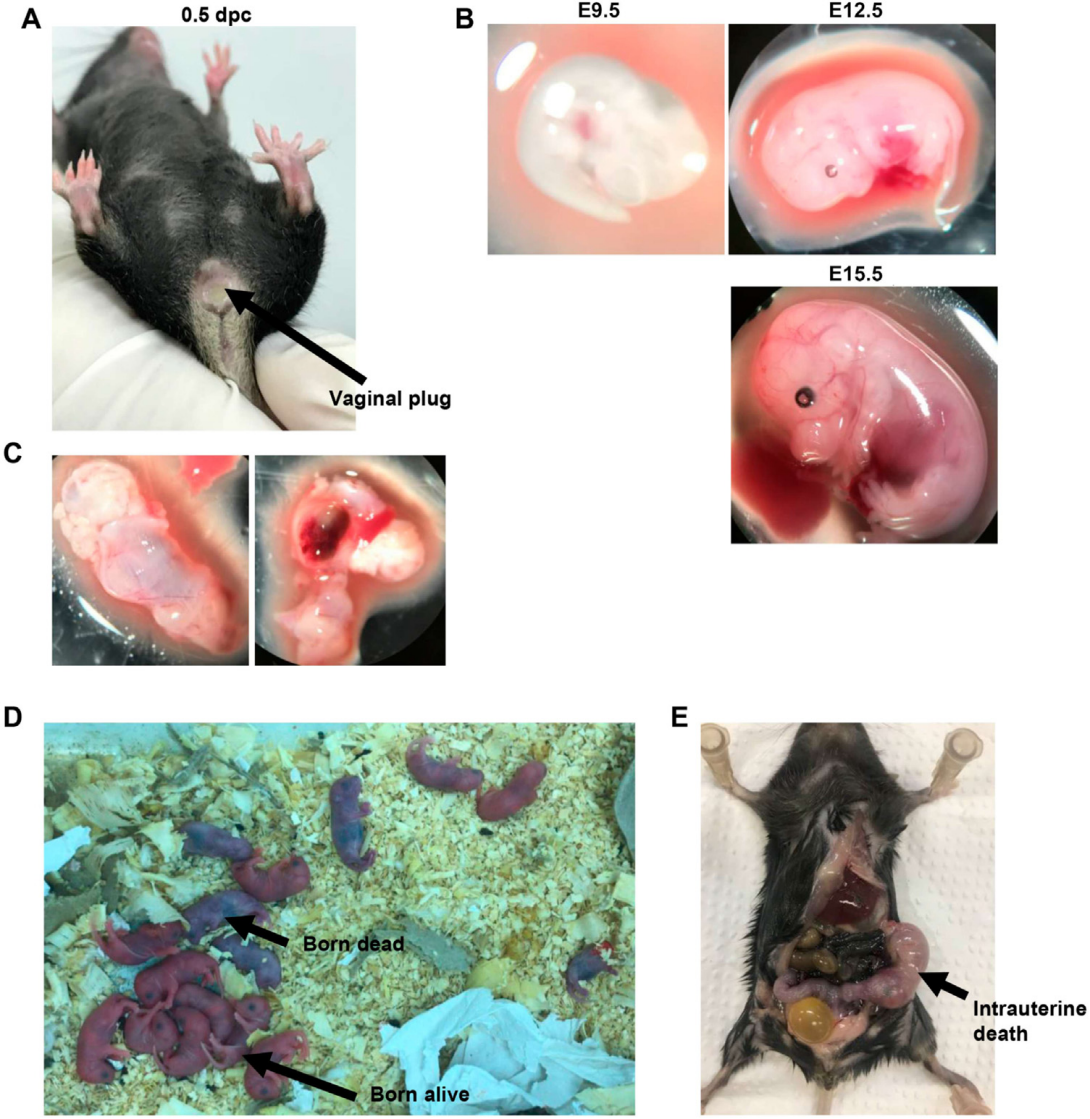
Evaluation of fetal development in Irs1^WT/S57X^ mice progeny. (A) Appearance of the vaginal plug; the arrow indicates the plug. (B) Visualization of fetuses at different gestational ages (E9.5; E12.5; E15.5); 10X magnification. (C) Evidence of fetal absorption in the uterus of a female at 15.5 days of gestation, 10X magnification. (D) Offspring of two females immediately after birth at E18.5. Both births resulted in one intrauterine death, six dead born and 11 born alive litters. (E) Intrauterine death in a postpartum female; the arrow indicates the fetus in the left uterine horn.

An evaluation of the progeny genotype at different gestational stages was performed to elucidate and prove the lethality of homozygous fetuses. A total of eight breeding pairs were established. The average time for plug identification in the evaluated females was 3.17 ± 0.98 days after the formation of mating partners. The presence of the plug indicates coitus, but does not guarantee pregnancy [15]. Euthanasia of the females was performed on gestational days E9.5 (*n* = 1), E12.5 (*n* = 1), E15.5 (*n* = 2), and E18.5 (*n* = 2). All females evaluated showed a positive plug (Figure 2A); however, one female sacrificed on day E9.5 and another on day E12.5 were not pregnant. Representative images of fetuses in different gestational phases can be seen in Figure 2B In each gestational phase, the total number of fetuses, the number of fetuses per uterine horn, the presence of signals of embryo absorption, and the genotype of the offspring were evaluated (Table 1). A female on E15.5 had only one fetus, with a homozygous genotype with signs of absorption being identified in this female (Figure 2C). Both females that would be sacrificed at E18.5 (at term) were maintained together, since the vaginal plug was identified on the same day. On the morning of the euthanasia day (E18.5), both females had given birth. There were no signs of breastfeeding of litters, indicating that the birth had occurred a few hours previously. Both deliveries resulted in 17 puppies, including 11 born alive and 6 aborted/died after birth (Figure 2D). After euthanasia, the females were dissected for verification, and one of them had a dead fetus in the left uterine horn (Figure 2E). The genotypes of the litters are described in Table 2.

**Table 1:** Description of progeny from Irs1WT/S57X crosses at different gestational stages.

Gestational age	E9.5	E12.5	E15.5 #1	E15.5 #2
				
**Fetuses**	9	10	7	1
**Right horn**	2	5	4	1
**Left horn**	7	5	3	0
**Absorption signs**	no	no	no	Yes
**Collected fetuses**	6	10	7	1
				
**Genotype**				
Irs1WT/WT	3	4	3	0
Irs1WT/S57X	3	2	3	0
Irs1S57X/S57X	0	4	1	1

D: day

WT/WT : Wild-type

WT/S57X : heterozygosis.

S57X/S57X : homozygous (Irs1 knockout phenotype).

**Table 2:** Characterization of newborn offspring from Irs1WT/S57X crosses.

D18.5 *n* = 2 females	Newborn mice	Irs1WT/WT	Genotype Irs1WT/S57X	Irs1S57X/S57X
Born alive	11	6	4	1
Born dead	6	0	0	6
Intrauterine death	1	0	1	0

D: day.

WT/WT : Wild-type.

WT/S57X : heterozygosis.

S57X/S57X : homozygous (Irs1 knockout phenotype).

## Discussion

Irs1-deficient mice are characterized by a reduction in growth, impaired glucose tolerance, and a decrease in glucose uptake [11,12]. In this study, the spontaneous truncated homozygosis mutation Irs1^S57X^, on a pure C57BL/6 genetic background, was associated with high fetal or postnatal mortality. In heterozygosis, the S57X mutation did not result in phenotypic changes. In contrast to these findings, Selman et al. described an increased lifespan in Irs1 null females compared to wild-type and heterozygotes [16]. Irs1-deficient models generated by targeted disruption do not exhibit significant fetal or postnatal lethality [11,12]. These data suggest that the elevated lethality observed in this study may not be directly related to Irs1 deficiency.

High lethality was described in a knockout model for *SerpinB2*, which spontaneously acquired the Irs1*^S57X^* mutation, resulting in the total loss of Irs1 protein expression and the *smla* phenotype [13]. Westrick et al. described a background sensitivity in the Irs1*^S57X^* model. Mice backcrossed to C57BL/6 J (at N3 and N4 generations) exhibit less than a 2 % survival rate at two weeks of age, unlike mice backcrossed to 129S1/SvImJ [13]. Genetic background has a relevant impact on phenotype alterations in the insulin receptor pathway [17,18].

In classical Mendelian inheritance, heterozygous intercrosses are expected to generate offspring in a 1:2:1 ratio. In the present study, chi-square analysis revealed a highly significant deviation from this expected distribution at birth, with a profound underrepresentation of homozygous Irs1^S57X^ animals. These findings indicate a markedly reduced perinatal survival associated with homozygosity. Although one homozygous animal survived until nine weeks of age, its extreme underrepresentation among offspring and the significant deviation from Mendelian proportions indicate that survival is rare and likely influenced by modifying genetic or environmental factors. Therefore, while complete lethality cannot be assumed, homozygosity for Irs1^S57X^ markedly compromises postnatal viability. Moreover, although the total number of offspring analyzed at birth was sufficient to detect statistical deviation, the number of pregnancies evaluated at specific embryonic stages was limited. Consequently, the precise developmental window responsible for lethality cannot be definitively established and warrants further investigation in larger cohorts.

An evaluation of the progeny of heterozygous pairs showed no significant implantation failure. Homozygous fetuses developed up to day of birth (E18.5), however, they were identified as aborted or died shortly after birth. Length of gestation is strain-dependent and normally lasts 19 to 21 days [15]. Based on these findings, further hematologic studies on B6.129S2-Irs1^smla^/J mice should focus on fetal hematopoiesis. This study was first developed to investigate the role of Irs1 in hematopoiesis, however, the absence of Irs1 knockout mice limited the expansion of hematological analysis between genotypes. The number of pregnant female mice at different gestational ages was also insufficient to perform statistical analysis to define an average number of genotypes in each stage. The results of the current study allowed the design of a new project for fetal hematopoiesis evaluation, since the Irs1 knockout fetuses develop until day E18.5.

## Conclusion

In summary, the Irs1^S57X^ mutation in heterozygous mice does not affect body weight or hematological parameters. In contrast, homozygosity for Irs1S57X is associated with markedly reduced perinatal survival, preventing studies of adult hematopoiesis. The findings indicate that lethality is not due to implantation failure, as homozygous fetuses develop until E18.5. These results highlight the limitations of this model for postnatal hematopoietic research. Future studies should focus on fetal hematopoiesis to elucidate the role of Irs1 in early hematopoietic development.

## Ethics approval

The study was approved by Animal Care and Use Committee of the Institution (CEUA/FMRP - Committee on Ethics in the Use of Animals at Ribeirão Preto Medical School).

## Data availability

The datasets generated and/or analyzed during the current study are available from the corresponding authors on reasonable request.

## CRediT authorship contribution statement

**Bruna Alves Fenerich:** Writing – original draft, Methodology, Investigation, Formal analysis, Data curation, Conceptualization. **Jaqueline Cristina Fernandes:** Investigation, Formal analysis, Writing – review & editing. **Cleide Lúcia Araújo Silva:** Investigation, Formal analysis, Writing – review & editing. **João Agostinho Machado-Neto:** Writing – original draft, Methodology, Investigation, Formal analysis, Data curation, Conceptualization. **Fabiola Traina:** Writing – original draft, Resources, Project administration, Funding acquisition, Data curation, Supervision, Conceptualization.

## Conflicts of interest

The authors declare no competing interests.

## References

[1] Machado-Neto J.A., Fenerich B.A., Rodrigues Alves A.P.N., Fernandes J.C., Scopim-Ribeiro R., Coelho-Silva J.L. (2018). Insulin substrate receptor (IRS) proteins in normal and malignant hematopoiesis. Clinics (Sao Paulo).

[2] Wang L.M., Myers M.G., Sun X.J., Aaronson S.A., White M., Pierce J.H (1993). IRS-1: essential for insulin- and IL-4-stimulated mitogenesis in hematopoietic cells. Science.

[3] Traina F., Carvalheira J.B., Saad M.J., Costa F.F., Saad S.T. (2003). BCR-ABL binds to IRS-1 and IRS-1 phosphorylation is inhibited by imatinib in K562 cells. FEBS Lett.

[4] Machado-Neto J.A., Favaro P., Lazarini M., Costa F.F., Olalla Saad S.T., Traina F. (2011). Knockdown of insulin receptor substrate 1 reduces proliferation and downregulates akt/mTOR and MAPK pathways in K562 cells. Biochim Biophys Acta.

[5] Rodrigues Alves A.P.N., Fernandes J.C., Fenerich B.A., Coelho-Silva J.L., Scheucher P.S., Simoes B.P. (2019). IGF1R/IRS1 targeting has cytotoxic activity and inhibits PI3K/AKT/mTOR and MAPK signaling in acute lymphoblastic leukemia cells. Cancer Lett.

[6] Khan M.Z., Zugaza J.L. (2025). Torres Aleman I. The signaling landscape of insulin-like growth factor 1. J Biol Chem.

[7] Artico L.L., Laranjeira A.B.A., Campos L.W., Correa J.R., Zenatti P.P., Carvalheira J.B.C. (2021). Physiologic IGFBP7 levels prolong IGF1R activation in acute lymphoblastic leukemia. Blood Adv.

[8] Fernandes J.C., Rodrigues Alves A.P.N., Machado-Neto J.A., Scopim-Ribeiro R., Fenerich B.A., da Silva F.B. (2017). IRS1/beta-catenin axis is activated and induces MYC expression in acute lymphoblastic leukemia cells. J Cell Biochem.

[9] Osorio F.G., Soria-Valles C., Santiago-Fernandez O., Bernal T., Mittelbrunn M., Colado E. (2016). Loss of the proteostasis factor AIRAPL causes myeloid transformation by deregulating IGF-1 signaling. Nat Med.

[10] Scopim-Ribeiro R., Machado-Neto J.A., Eide C.A., Coelho-Silva J.L., Fenerich B.A., Fernandes J.C. (2021). NT157, an IGF1R-IRS1/2 inhibitor, exhibits antineoplastic effects in pre-clinical models of chronic myeloid leukemia. Invest New Drugs.

[11] Araki E., Lipes M.A., Patti M.E., Bruning J.C., Haag B., Johnson R.S. (1994). Alternative pathway of insulin signalling in mice with targeted disruption of the IRS-1 gene. Nature.

[12] Tamemoto H., Kadowaki T., Tobe K., Yagi T., Sakura H., Hayakawa T. (1994). Insulin resistance and growth retardation in mice lacking insulin receptor substrate-1. Nature.

[13] Westrick R.J., Mohlke K.L., Korepta L.M., Yang A.Y., Zhu G., Manning S.L. (2010). Spontaneous Irs1 passenger mutation linked to a gene-targeted SerpinB2 allele. Proc Natl Acad Sci U S A.

[14] Wong M.D., van Eede M.C., Spring S., Jevtic S., Boughner J.C., Lerch J.P. (2015). 4D atlas of the mouse embryo for precise morphological staging. Development.

[15] Coleman D.L., Kaliss N., Dagg C.P., Russell E.S., Fuller J.L., Staats J. (1966).

[16] Selman C., Lingard S., Choudhury A.I., Batterham R.L., Claret M., Clements M. (2008). Evidence for lifespan extension and delayed age-related biomarkers in insulin receptor substrate 1 null mice. FASEB J.

[17] Kulkarni R.N., Almind K., Goren H.J., Winnay J.N., Ueki K., Okada T. (2003). Impact of genetic background on development of hyperinsulinemia and diabetes in insulin receptor/insulin receptor substrate-1 double heterozygous mice. Diabetes.

[18] Kido Y., Philippe N., Schaffer A.A., Accili D. (2000). Genetic modifiers of the insulin resistance phenotype in mice. Diabetes.

